# TGF-β signaling regulates differentiation of MSCs in bone metabolism: disputes among viewpoints

**DOI:** 10.1186/s13287-024-03761-w

**Published:** 2024-05-31

**Authors:** Erfan Wei, Menglong Hu, Likun Wu, Xingtong Pan, Qiyue Zhu, Hao Liu, Yunsong Liu

**Affiliations:** 1 Department of Prosthodontics, Peking University School and Hospital of Stomatology & National Center for Stomatology & National Clinical Research Center for Oral Diseases & National Engineering Research Center of Oral Biomaterials and Digital Medical Devices& Beijing Key Laboratory of Digital Stomatology & NHC Key Laboratory of Digital Stomatology & NMPA Key Laboratory for Dental Materials, Central Laboratory, Peking University School and Hospital of Stomatology , No.22, Zhongguancun South Avenue, Haidian District, Beijing, 100081 PR China; 2 Central Laboratory, Peking University School and Hospital of Stomatology & National Center for Stomatology & National Clinical Research Center for Oral Diseases & National Engineering Research Center of Oral Biomaterials and Digital Medical Devices& Beijing Key Laboratory of Digital Stomatology & NHC Key Laboratory of Digital Stomatology & NMPA Key Laboratory for Dental Materials , Peking University School and Hospital of Stomatology, No.22, Zhongguancun South Avenue, Haidian District, Beijing, 100081 PR China

**Keywords:** TGF-β, MSCs, Osteogenic differentiation, Bone metabolism

## Abstract

Mesenchymal stem cells (MSCs) are multipotent cells that can differentiate into cells of different lineages to form mesenchymal tissues, which are promising in regard to treatment for bone diseases. Their osteogenic differentiation is under the tight regulation of intrinsic and extrinsic factors. Transforming growth factor β (TGF-β) is an essential growth factor in bone metabolism, which regulates the differentiation of MSCs. However, published studies differ in their views on whether TGF-β signaling regulates the osteogenic differentiation of MSCs positively or negatively. The controversial results have not been summarized systematically and the related explanations are required. Therefore, we reviewed the basics of TGF-β signaling and summarized how each of three isoforms regulates osteogenic differentiation. Three isoforms of TGF-β (TGF-β1/β2/β3) play distinct roles in regulating osteogenic differentiation of MSCs. Additionally, other possible sources of conflicts are summarized here. Further understanding of TGF-β signaling regulation in MSCs may lead to new applications to promote bone regeneration and improve therapies for bone diseases.

**Supplementary Information:**

The online version contains supplementary material available at 10.1186/s13287-024-03761-w.

## Introduction

Bone tissues are essential in the human body for mechanical and metabolic functions [1]. Bone formation and resorption are dynamically balanced in a healthy skeleton, and dysregulation of the process can lead to metabolic diseases [2]. Mesenchymal stem cells (MSCs) are stem cells with the potential to differentiate into multiple forms of mesenchymal tissue, including bone tissue. MSCs have drawn much attention in regard to the treatment of bone diseases and defects, due to their pluripotency, abundant sources, and general lack of ethical problems [3, 4]. Differentiation patterns of MSCs are affected by multiple intrinsic and extrinsic factors, and the osteogenic differentiation of MSCs relates to the expression of multiple genes in a particular sequence under tight regulation [5, 6].

Transforming growth factor β (TGF-β) is a member of the TGF-β superfamily, which includes more than 30 secreted growth factors [1]. TGF-β signaling plays an essential role in many biological processes, which include development [7], immunoregulation [8], cancer progression [9], cardiovascular disease [10], bone development [11] and so on. Besides, TGF-β is also an important factor that regulates the functions of MSCs in bone metabolism [1]. Disruption of TGF-β signaling in bone metabolism underlies congenital defects, acquired diseases, and defective healing of bone tissue [12, 13]. Confusingly, the conflict viewpoints of TGF-β signaling in osteogenic differentiation of MSCs exit among some of in vitro and in vivo studies. Loss of TGF-β receptor 1 (TR1) in mice primary neonatal calvarial cells leads to inhibition of osteogenic differentiation [14]. Mesenchymal cells from mice embryo skull displayed decreased osteogenic differentiation after knockout of TGF-β receptor 2 (TR2), which is supported by down regulated expression of *runt-related transcription factor 2* (*Runx2*), *osterix* (*Osx*), *distal-less homeobox gene* (*Dlx5*) and *Msh homeobox 2* (*Msx2*) [15]. We can know that TGF-β signaling is necessary for osteogenic differentiation of MSCs according to the above reports. However, Li et al. showed that TGF-β1 treatment inhibited osteogenic differentiation of mesenchymal progenitor cells (MPCs) [16]. Chen et al. also reported high TGF-β1 in periodontitis reduced osteogenic differentiation of MSCs recently [17]. These results may seems contradict to the conclusion above and the controversial results may not been summarized and fully explained in existing literature. Therefore, it is necessary to summarize how TGF-β signaling regulates osteogenic differentiation of MSCs and try to explain the possible sources of this conflicts.

Here, we searched the published studies focusing on roles of TGF-β in osteogenic differentiation systematically, and the related reviews are searched as well. The disputes in viewpoints mentioned above and possible explanations has not been well summarized in published literature. It is also found that three TGF-β isoforms tend to play distinct roles in osteogenic differentiation, which is less reviewed in published literature. Therefore, we review the mechanisms of the TGF-β signaling pathways and summarized the roles of three TGF-β isoforms in regulating MSCs in osteogenic differentiation respectively. Different TGF-β isoforms play different role in osteogenic differentiation of MSCs. Possible sources of contradictory viewpoints in published studies are also summarized in here. We want to provide a more detailed map of TGF-β signaling in osteogenic differentiation of MSCs and hope to inspire potential therapies for bone diseases and defects in the future.

## Methods

### Objectives

The objectives are to review the researches focusing on regulation of osteogenic differentiation of MSCs in vitro and bone formation in vivo by three TGF-β isoforms, to provide an overview of the controversial topic and inspire further studies on application and mechanisms of TGF-β isoforms in bone regeneration in future.

### Inclusion and exclusion criteria of literature

#### Inclusion criteria of literature

- In vitro studies that analyze the role of TGF-β1,2,3 in osteogenic differentiation of MSCs or primary cells of mesenchymal origin.
- In vivo studies that analyze the role of TGF-β1,2,3 in bone formation with bone-defect animal models.

#### Exclusion criteria of literature

- Reviews, books, meetings, patents, letters and literature updates.
- Unclear statement of which TGF-β isoform is discussed in the study.
- Studies with no control group.
- Articles without full text.

### Search strategy

When reviewing the literature, the Pubmed, Embase, Web of Science and Chinese Medical Literature databases are searched for in vivo and in vitro studies investigating roles of TGF-β1,2,3 in osteogenic differentiation of MSCs up to September 2023. The Mesh words “TGF-β1”, “TGF-β2”, “TGF-β3” and “osteogenesis” are included separately, and the details are presented in Appendix 1. The same searching words are used to searching for published reviews on the topic.

### Study selection

**Fig. 1:**
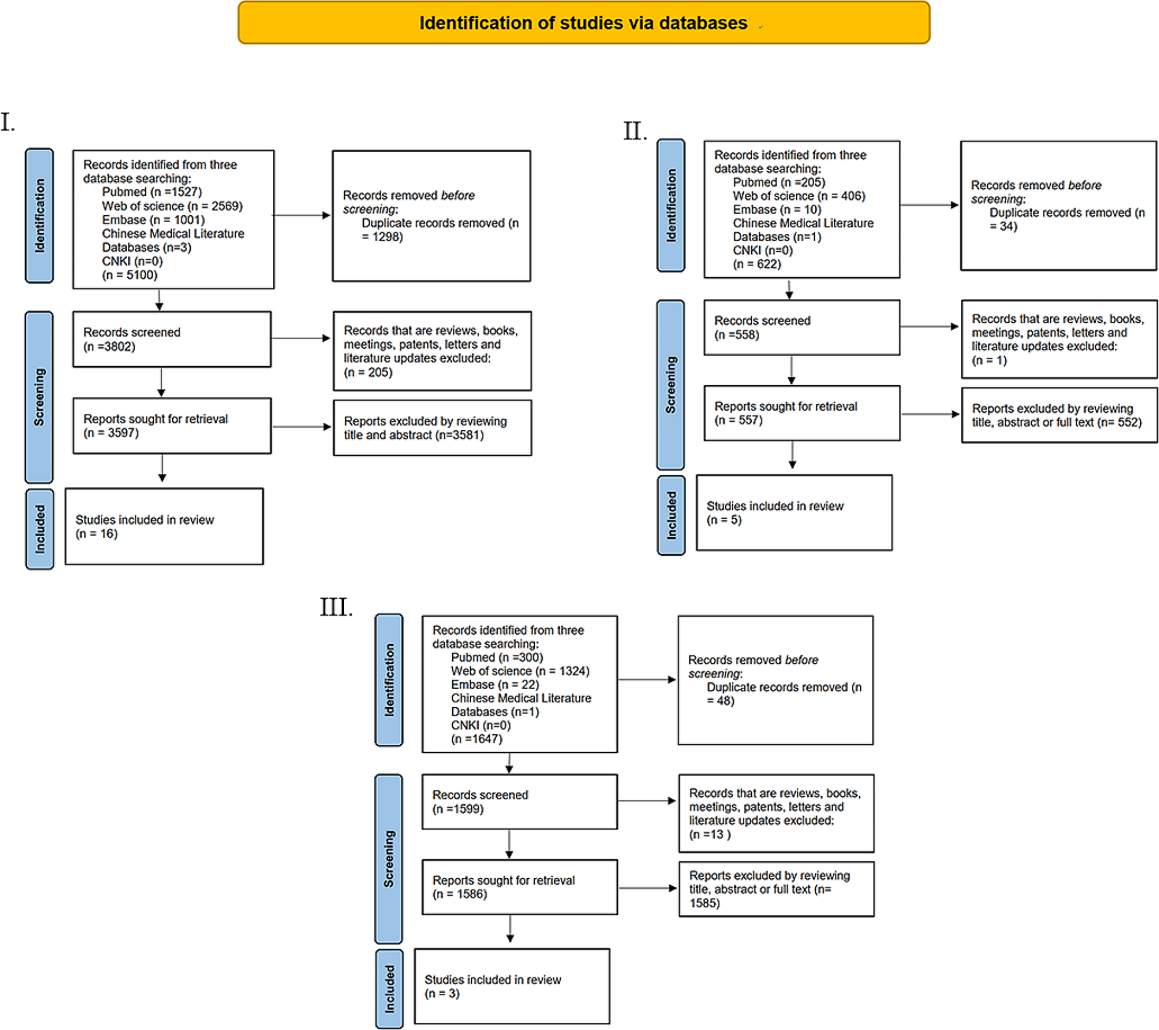
Flow charts of the studies selection process. **I**. **II**. and **III**. represents selection process for three isoforms TGF-β1, 2 and 3 separately. The template of flow charts is referred to PRISMA (http://www.prisma-statement.org)

Studies were selected by two reviewers (Wei E and Hu M) independently, and disagreements were solved by consultation and discussion with the third reviewer (Liu Y). The titles and abstracts are reviewed firstly, and full text of the retrieved studies are accessed in reference to the inclusion and exclusion criteria mentioned above. Flow charts of the select process are shown in Fig. 1. For the searching of reviews on this topic, 92 reviews are searched and the tittles and abstracts are firstly screened. Full text of 14 reviews are read carefully.

## The basics of TGF-β signaling

Mammals have three TGF-β isoforms, TGF-β1, TGF-β2, and TGF-β3. They also have two serine/threonine kinase receptors, including TR1 and TR2. Betaglycan, also known as TR3, was originally identified as a coreceptor [18] but more functions have been revealed since [19–21]. TGF-βs bind with receptors on the cytomembrane and activate intracellular signaling to regulate particular gene expression [22].

### Structure of latent TGF-β and its activation

**Fig. 2:**
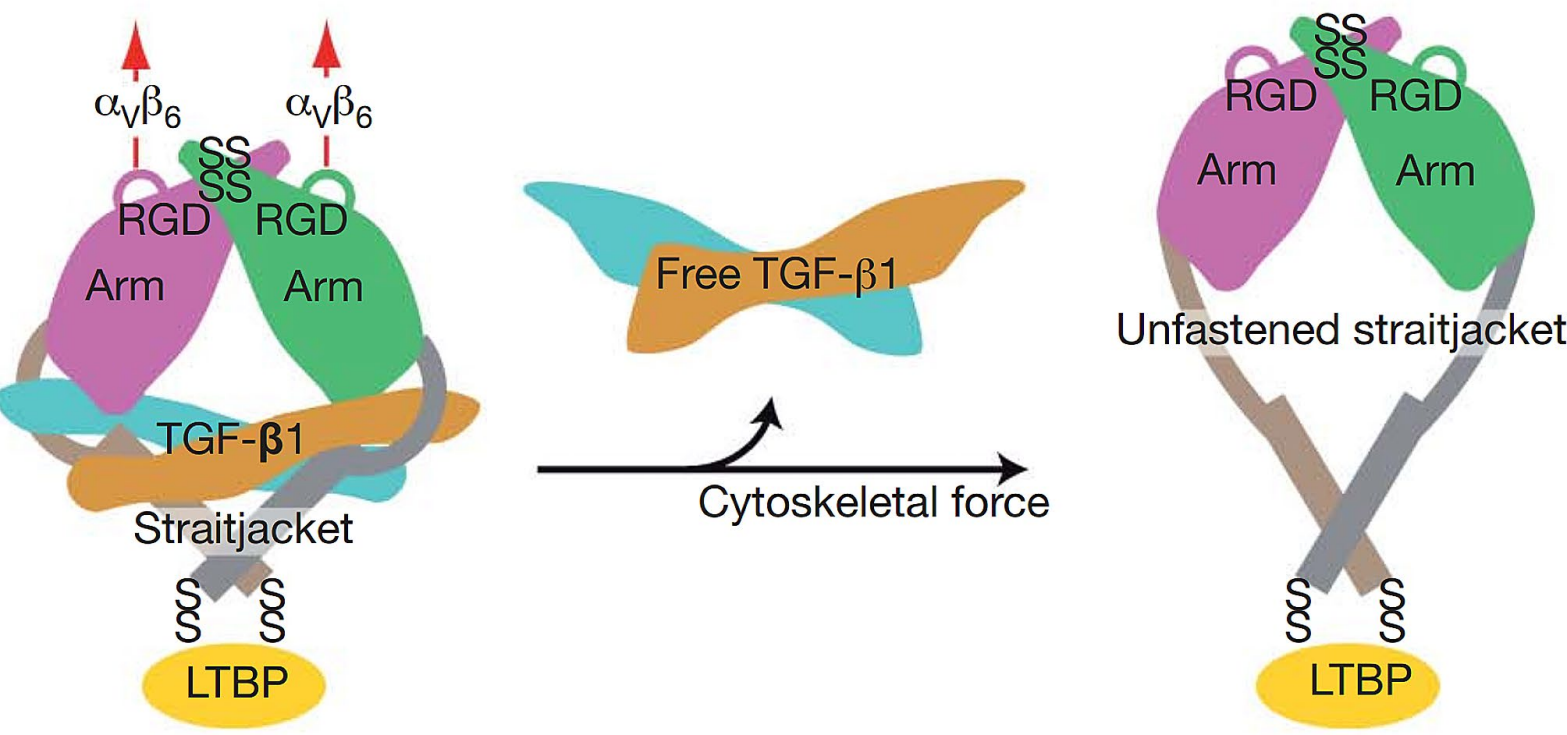
Schematic of the large triple complex (LCC) and its activation mechanisms by αVβ6 integrin. The prodomain consists of an arm domain and a straitjacket. Two prodomains noncovalently bond with a TGF-β homodimer. Activation of TGF-β requires the binding of αV integrin with the RDG sequence (Arg-Gly Asp) in the prodomain and the exertion of force on this domain. After the straitjacket is unfastened, the TGF-β dimer is released and can bind with receptors. SS, disulfide bonds.

TGF-βs are synthesized as large precursors with an amino-terminal prodomain in ribosomes attached to the endoplasmic reticulum (ER). The dimerization of TGF-β monomers occurs in the ER via disulfide bonds. The pro-TGF-β dimers crosslink with the latent TGF-β binding proteins (LTBPs) in the ER, forming a large latent complex (LLC). In the trans-Golgi, the amino prodomain of the TGF-β, also known as the latent associated peptide (LAP), is cleaved, and the cleaved LLC is secreted via exocytosis [23]. TGF-βs mostly exist as disulfide-linked homodimers with a cysteine-rich TGF-β knot in each monomer after secretion [24]. In addition to homodimers, the heterodimers TGF-β2/3 and TGF-β1/2 have also been isolated from the bones of cattle [25]. TGF-βs are stored in the extracellular matrix (ECM) as latent forms, and LAP sequesters them from their receptors. LAP often exists in complexes with LTBPs (Fig. 2) [26].

Furthermore, activation of latent TGF-βs depends on proteases and integrins [23]. Structure analysis of the latent TGF-β complex has revealed that TGF-β1 activation by αV integrin occurs through a conformational change in LAPs in a force-dependent manner. The similarity in prodomain folding in the TGF-β family members indicates a similar activation mechanism (Fig. 2) [26].

### Interaction between ligands and receptors

**Fig. 3:**
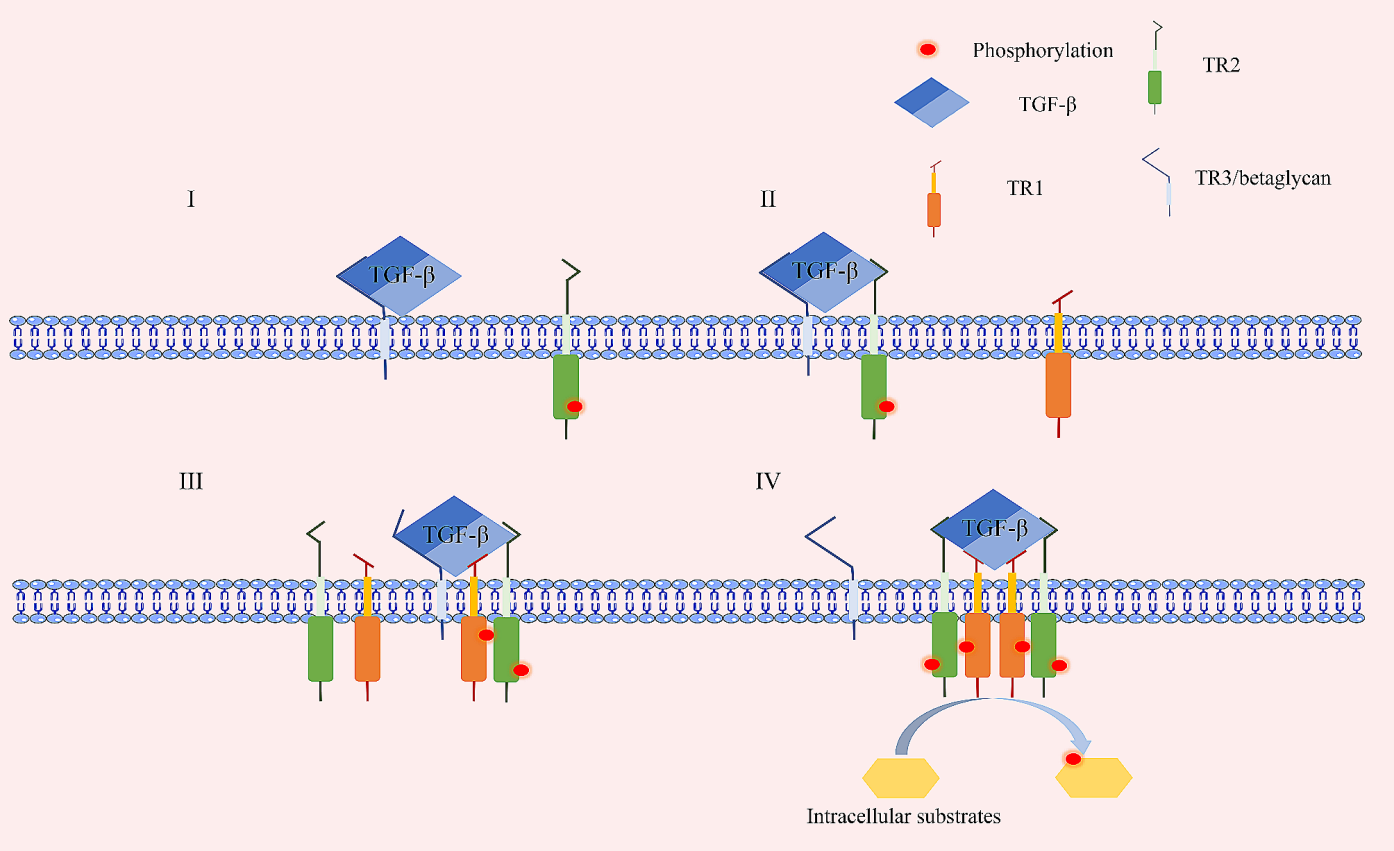
Interaction between TGF-β ligands and receptors. (**I**) TGF-β dimer (blue parallelogram) first binds with betaglycan (blue receptor) with 1:1 stoichiometry and is potentiated to bind to TR2 (green receptor). One modular of TR2 is allowed to bind with the complex. TR2 is constitutively phosphorylated (red circles) independent of ligands. (**II**) TR2 binds with the ligands and potentiates TR1 (orange receptor) to interact with the TGF-β/TR2 complex. **(III)** TR1 binds to the complex and stabilizes the interaction between TR2 and the ligand. (**IV**) TR1 is phosphorylated by TR2 and activated to phosphorylate (red circles) downstream substrates (yellow parallelogon). However, betaglycan must be displaced for TR1 binding. Betaglycan is totally displaced due to lower affinity caused by the modified binding state with the TGF-β dimer. Finally, a ligand–receptor complex consisting of a TGF-β homodimer, a pair of TR2, and a pair of TR1 is formed.

TR1 is a transmembrane glycoprotein made up of an extracellular TGF-β binding domain, a single transmembrane domain, and an intracellular serine/threonine kinase domain [27]. The overall structure of TR2 is like that of TR1 [28]. Besides, TR1 contains a glycine- and serine-rich domain (GS domain) preceding the kinase domain, which is essential for TGF-β signaling [27]. Wrana et al. revealed the interaction mechanisms between TGF-β ligands and receptors (Fig. 3). TR2 is constitutively phosphorylated by cellular kinases and itself at different sites independent of ligand binding. TR2, but not TR1, displays a high affinity for TGF-βs, and TR1 only interacts with high affinity with the TGF-β/TR2 complex [29]. Dimeric TGF-β binds to the extracellular domain of TR2, and TR1 recognizes the complex; it recruits TR1 to form the ligand–receptor complex, which consists of a TGF-β homodimer, a pair of TR2s, and a pair of TR1s [30]. However, the affinity between TR1 and TR2 is low without ligands. TR2 phosphorylates TR1 in the complex in the GS domain and phosphorylates downstream substrates [29]. The different kinetics and structural differences in the receptor binding of the three TGF-β isoforms may underlie their unique biological effects [30].

Betaglycan (TR3) functions as a coreceptor in TGF-β signaling. Its core protein is a transmembrane protein consisting of an amino-terminal ectodomain, a transmembrane domain, and a short cytoplasm tail [31]. Betaglycan is hypothesized to function as a coreceptor through a “handoff” mechanism in which it first binds with the ligand, thus potentiating the ligand to bind to TR2 (Fig. 3). Then the binding of TR2 recruits TR1 to displace the betaglycan partially, and TR1 stabilizes the weakly bound TR2 through receptor–receptor contact [32, 33]. The ectodomain can be released as a soluble proteoglycan, which binds with TGF-βs and functions as an antagonist [19]. The TGF-β1 and TGF-β3 isoforms show high affinity for TR2 and can assemble a signaling complex without betaglycan. However, TGF-β2 displays a much lower affinity for TR2, and betaglycan is essential to render TGF-β2 as potent as TGF-β1 [30, 34]. In addition, the cytoplasmic domain of TR3 interacts with scaffold proteins (mainly Gα-interacting protein-interacting protein C-terminus, GIPC) and competes for binding with TR1 and TR2 separately, thus inhibiting TGF-β signaling [21]. However, for epicardial cells to be responsiveness to TGF-β1 and TGF-β2, it is essential for TR3 to bind with GIPC1 [20]. The functions of betaclycan are distinct in different cells, and its exact functions require further exploration.

### Canonical signaling of TGF-β

**Fig. 4:**
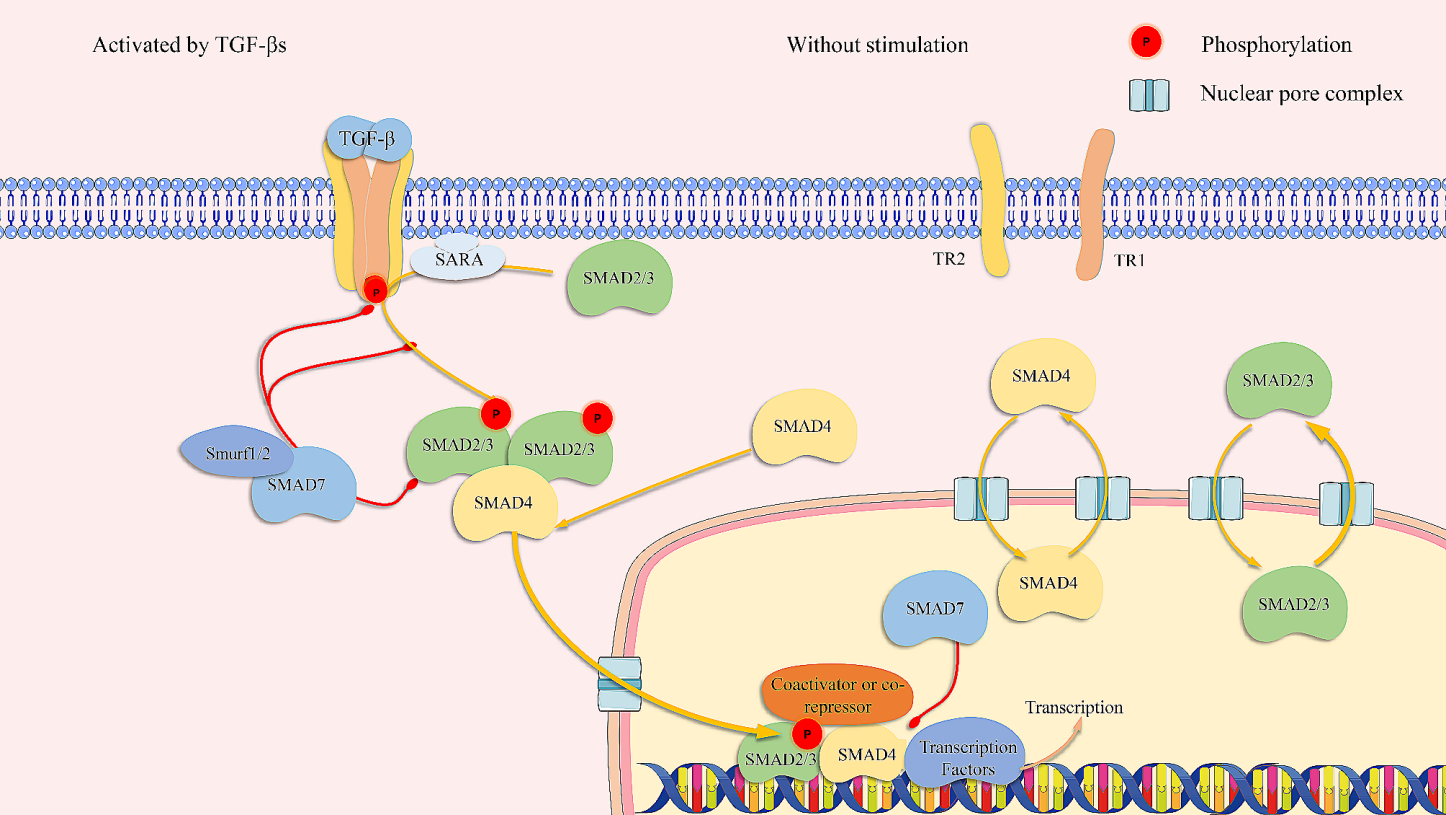
Canonical signaling of TGF-β. SMAD2 or SMAD3 can be activated by TR1 phosphorylation (red circles). Activated SMAD2/3 form SMAD complexes with SMAD4, which are transported into the nucleus to regulate gene expression. SMAD proteins undergo nucleoplasm shuttling without stimulation.

*Sma* and *Mad* homolog (SMAD) proteins are well-recognized intracellular mediators of TGF-β signaling. Intracellular signaling depending on SMAD carboxy-terminal phosphorylation by TR1 is termed “canonical signaling” (Fig. 4) [18, 35]. Only receptor-activated SMADs (R-SMADs), including SMAD1, 2, 3, 5, and 8, can interact directly with receptors of the TGF-β superfamily. The R-SMADs are selectively activated depending on which type I receptors are activated by the ligands of TGF-β superfamily. SMAD2 and SMAD3 are activated through carboxy-terminal phosphorylation by TGF-β and actin family receptors. SMAD 1,5,8 are mainly activated by receptors of BMPs and other members of the TGF-β superfamily. The remaining classes of SMADs include co-SMAD, namely SMAD4, and inhibitory SMADs (I-SMADs). I-SMADs include SMAD6 and SMAD7. SMAD proteins are greatly conserved among different species. R-SMADs and SMAD4 harbor an amino-terminal Mad-homology 1 (MH1) domain and a carboxy-terminal Mad-homology 2 (MH2) domain, linked by a less well-conserved linker region. Functionally, the MH1 domain is related to nuclear localization and DNA binding (except in SMAD2), whereas the MH2 domain is related to receptor interaction and oligomerization of SMADs [35, 36].

The SMAD anchor for receptor (SARA) is essential for responding to TGF-β signaling. It specifically recruits unphosphorylated SMAD2 and SMAD3 to the TGF-β receptor, where SMAD2 is released once phosphorylated [37]. Once activated by TR1, SMAD2 and SMAD3 are phosphorylated and oligomerized with SMAD4. The SMAD proteins undergo nucleoplasm shuttling without stimulation, where the export rate of the activated SMAD complex from the nucleus is decreased, and the import rate into the nucleus is increased. The complex is thus trapped in the nucleus [38]. The SMAD proteins regulate transcription in a context-dependent manner by binding to gene enhancers or promoters enriched in 5 bp GC-rich sites (5GC SMAD binding elements) and cooperating with other transcription factors and transcriptional coactivators or corepressors in different contexts [39]. CREB binding protein (CBP), p300, and P/CAF are coactivators of SMAD-induced transcription in multiple cell lines [40, 41].

Inhibitory SMADs have a conserved carboxy-terminal MH2 domain that interacts with receptors and SMADs. SMAD6 is believed to inhibit SMAD signaling induced by BMP preferentially, but SMAD7 inhibits SMAD signaling induced by TGF-β and BMP equally. ISMs function through multiple mechanisms, including interfering with the activation of RSMs by the receptors, inducing degradation of receptors, and interfering with transcription regulated by the SMAD complex [42]. In addition, SMAD7 completes with R-SMAD-SMAD4 oligomerization and recruits E3 ubiquitin ligase to mediate polyubiquitination and degeneration of activated R-SMADs [43].

In summary, SMADs are intracellular mediators of TGF-β. In canonical signaling of TGF-β, SMAD2 and SMAD3 can be phosphorylated by TR1, and they oligomerize with SMAD4. The SMAD complexes are transported into the nucleus and bind to DNA or interact with other transcription factors. I-SMADs can interrupt TGF-β signaling by interacting with receptors or other SMADs (Fig. 3).

### Noncanonical signaling of TGF-β

**Fig. 5:**
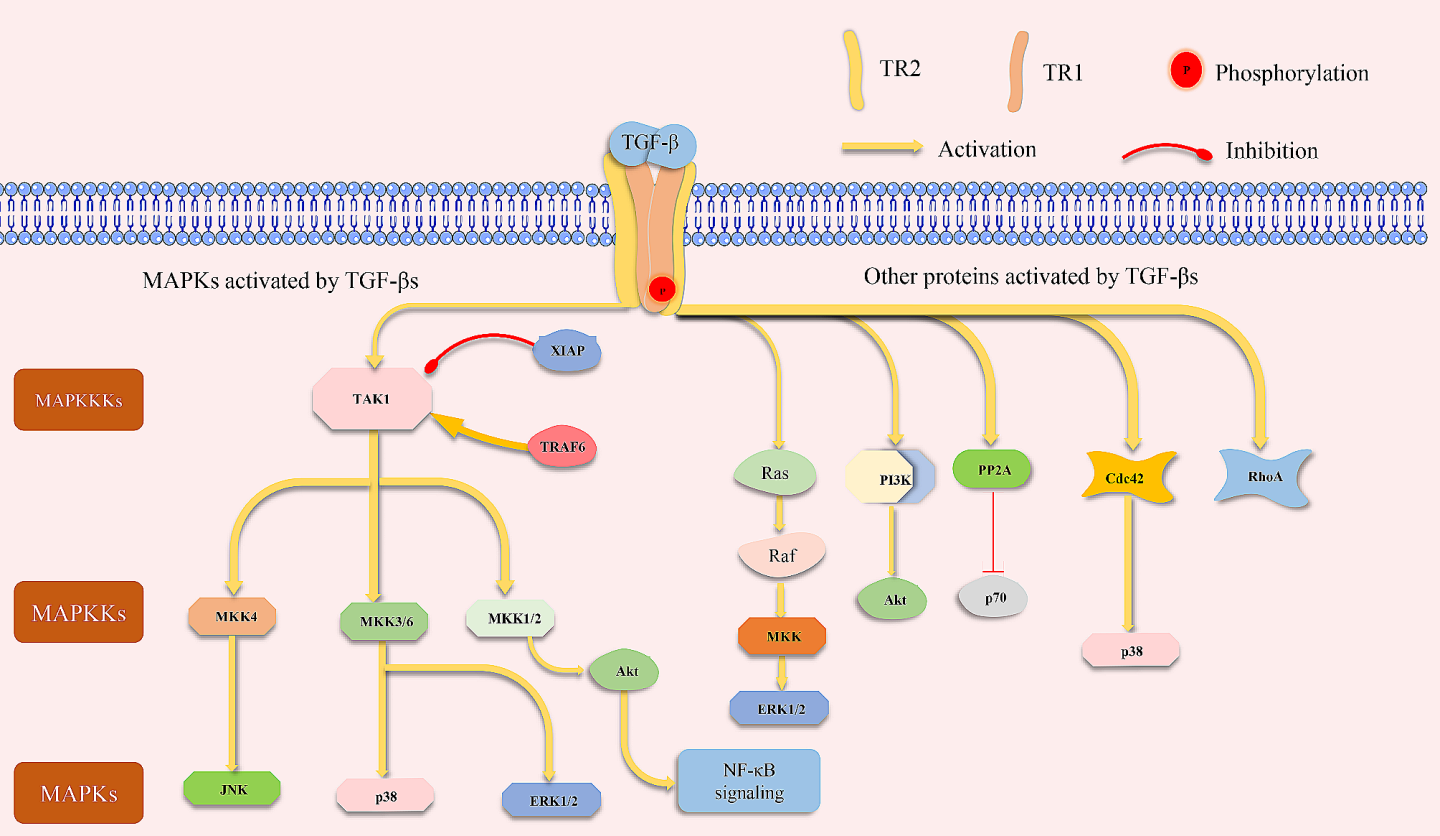
Noncanonical signaling of TGF-β. TGF-βs can activate many other intracellular pathways independent of the SMAD proteins, including MAPKs, Rho-like GTPases, and PI3K. (Red circle: phosphorylation of TR1).

In addition to canonical signaling of TGF-β, “noncanonical signaling” also occurs, namely, SMAD-independent downstream pathways of TGF-β signaling [23] (Fig. 5).

TGF-βs can activate mitogen-activated protein kinases (MAPKs) independent of SMADs [23]. MAPKs are intracellular modules activated by extracellular stimuli. There are five distinct groups: extracellular signal-regulated kinases (ERKs) 1 and 2; c-Jun amino-terminal kinases (JNKs) 1, 2, and 3; p38 isoforms; and ERK 3, 4, and 5, each of which include three sequentially acting kinases, namely, a MAPK, a MAPK kinase (MKK), and a MAPKK kinase (MKKK) [44]. TGF-β-activated kinase 1 (TAK1) is one type of MKKK that is activated by TGF-β or BMP [45]. MAPKs can be activated by TGF-β independent of SMAD [23]. TAK1 can also activate MKK3 and MKK6, thus activating downstream p38 and regulating the expression of *Runx2* [46]. TAK1 activated by TGF-β interacts with MKK1/2 and activates the AKT/NF-κB pathway [47]. Ubiquitination of TAK1 by the X-linked inhibitor of apoptosis protein (XIAP) causes proteolysis of TAK1 and inhibition of JNK1 downstream [48]. Tumor necrosis factor (TNF) receptor-associated factor (TRAF) is essential for the activation of JNK and p38 by TGF-β [49].

Except for MAPKs, other proteins such as Rho-like GTPases, phosphatidylinositol-3-kinase (PI3K), and protein phosphatase 2 A (PP2A) also interact with TGF-β receptors independent of SMADs [45]. Rho-like GTPase family members including RhoA, RhoB, Cdc42, and Rac1 are important in various signaling events [35]. TGF-β1 can activate RhoA and Cdc42, and p38 is activated by Cdc42 [50]. PI3K activated by TGF-β reportedly activates AKT, followed by the mammalian target of rapamycin (mTOR) [51].

In summary, TGF-β regulates biological processes through a complex and multi-step process, which is shown in Fig. 7. TGF-β ligands bind to receptors on the cytoplasm and form a ligand–receptor complex. SMADs are intracellular substrates of TR1, but SMAD-independent signaling pathways also exist, also known as the noncanonical signaling of TGF-β. SMADs or other intracellular proteins activated by TGF-β signaling function as transcription factors or regulate other transcription factors, ultimately controlling gene expression. With further investigations in TGF-β, we will achieve a more comprehensive understanding of structure and function of TGF-β.

## Role of TGF-β signaling in MSC functions in bone metabolism – conflicting viewpoints

TGF-β signaling plays an essential role in cell fate determination of MSCs. MSCs can differentiate into osteoblasts, and many modular factors tightly regulate the process. Furthermore, MSCs can differentiate into common osteo-chondroprogenitor cells, committed osteoprogenitor cells, and pre-osteoblasts, and a series of osteogenic genes are expressed under tight regulation in sequence [5].

TGF-βs are indispensable factors in regulation of osteogenesis. In healthy adult bone, the dynamic balance between bone formation and bone resorption is maintained [1]. However, dysregulation of the balance may lead to osteoporosis, osteosclerosis, and other bone diseases [2]. Studies shown that all three TGF-β isoforms are expressed in bone tissue; however, the basal level of TGF-β1 expression is significantly higher than the other isoforms as measured by mRNA levels in mouse tibial diaphysis. Besides, the basal expression level of TGF-β2 is higher than that of TGF-β3 [52]. TGF-β signaling pathway plays an important role in the osteogenic differentiation of MSCs, but whether it promotes or inhibits osteogenic differentiation is controversial [17, 53, 54]. To further search possible explanations for conflicts on the topic, published reviews on role of TGF-β signaling in osteogenic differentiation or bone formation are searched. It is found that the controversial viewpoints and possible explanations have not been fully discussed by published literature. Although it has been summarized that TGF-β signaling promotes osteogenic differentiation in an early stage but inhibits maturation of osteoblasts [1], this may not fully explain the distinct roles of TGF-βs in cells with no markable difference in differentiation stage and the conflict results of in vivo studies [55–61]. Therefore, a systematic summary and searching for possible explanations on the topic may still be necessary to provide reference for future applications.

**Fig. 6:**
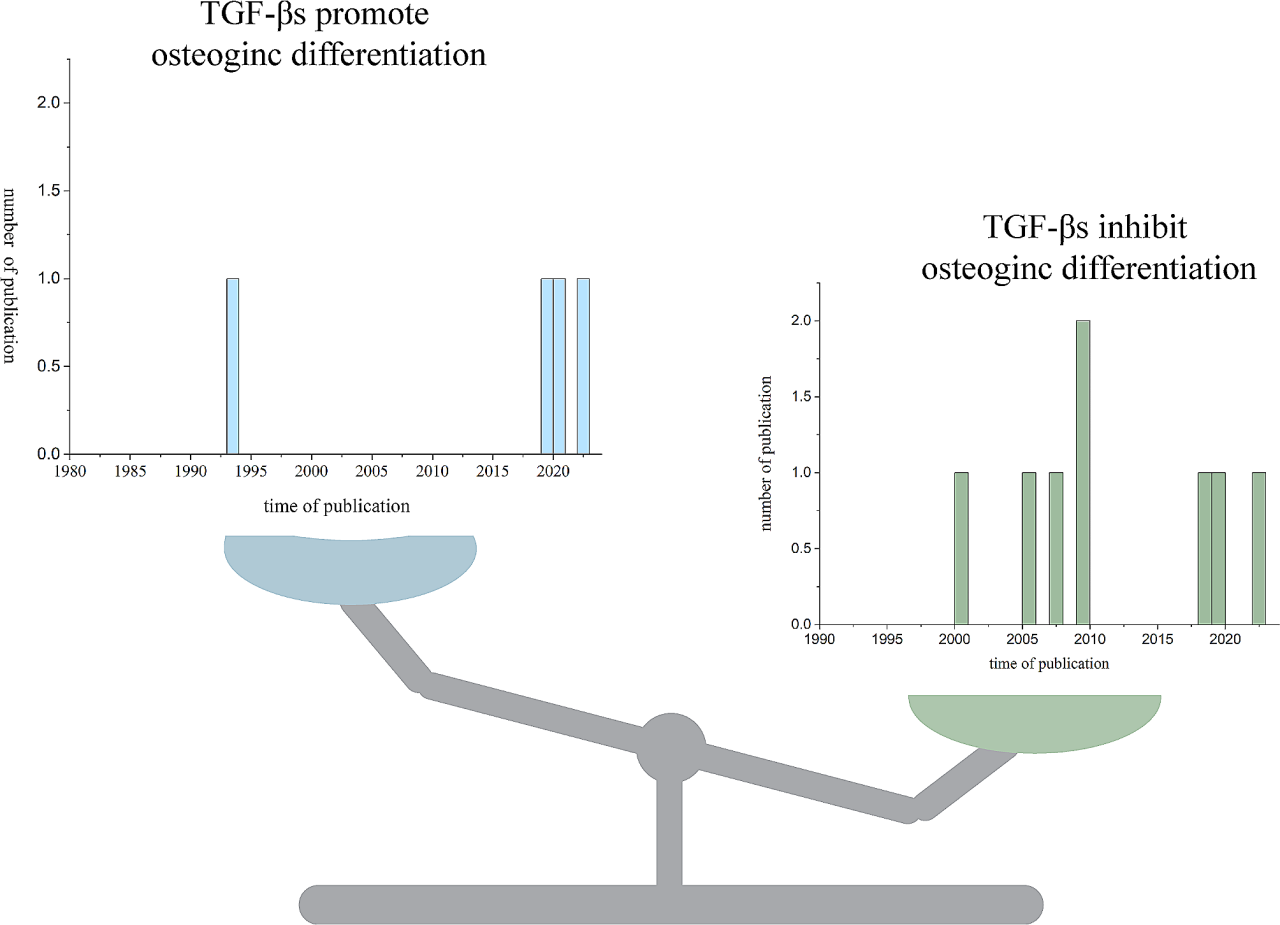
The distribution of literature that supports promotion or inhibition of TGF-β signaling in osteogenic differentiation of MSCs. Considering number of studies, more studies tend to support that TGF-β signaling displays an inhibition role in osteogenic differentiation of MSCs. During 2000–2012, more studies tended to support that TGF-β signaling inhibits osteogenic of MSCs. However, studies that support both views are almost equal in recent years.

The distribution of literature that supports promotion or inhibition of TGF-β signaling in osteogenic differentiation of MSCs is displayed in Fig. 6. All the included studies are considered. During 2000–2012, more studies tended to support that TGF-β signaling inhibits osteogenic of MSCs. However, studies that support both views are almost equal in recent years. Interestingly, considering number of studies, more studies tend to support that TGF-β signaling displays an inhibition role in osteogenic differentiation of MSCs, which is also not completely consist with existing views [1, 62, 63]. Therefore, a systematic searching and review of literature on this topic may be required.

In the included studies, it is found that the different TGF-β isoforms may play distinct roles in osteogenic differentiation of MSCs, and we try to discuss the possible explanations underlying the different viewpoints that are based on previous researches.

### TGF-β1 regulates functions of MSCs

There is accumulating evidence supporting the idea that TGF-β1 regulates MSCs functions and bone formation [16, 53, 54, 59, 64, 65]. However, it is not clear whether TGF-β1 promotes or suppresses osteogenesis in vitro. Li et al. reported that TGF-β1 promotes osteogenic differentiation of human bone marrow stromal cells (BMSCs). The conclusions were proven by *OCN* and *RUNX2* expression in BMSCs overexpressing TGF-β1 and the control group on days 7 and 14 and alizarin red staining (ARS) on day 14 [54]. However, Kwok et al. reported that TGF-β1 treatment inhibited the expression of osteoblast differentiation genes, including *alkaline phosphatase* (*Alp*) and *Osteocalcin* (*Ocn*), and mineralization in differentiating osteoblasts of rats, which disagrees with Li et al. However, the expression of *Runx2* was not changed by TGF-β1 treatment [53]. The controversial results above may be attributed to distinct response of TGF-β1 of distinct species. Li et al. also showed that mesenchymal progenitor cells (MPCs) from 3- and 9-month-old mice were inhibited from differentiating into osteoblast after TGF-β1 pretreatment. This was proven by significantly reduced ALP^+^ cells compared to the control [16]. Zhang et al. found that TGF-β1 inhibited osteogenic differentiation and promoted premature senescence of BMSCs under insulin treatment, but osteogenic differentiation of BMSCs without insulin treatment are not changed significantly by TGF-β1 [64]. Therefore, some specific conditions, for example, a high insulin environment, may have influence on response of BMSCs to TGF-β1 and may change their osteogenic differentiation in response to TGF-β1.

In in vivo studies, TGF-β1 has been found to promote bone formation. Intravenous injection of TGF-β1 into rats and rabbits at 1000 µg/kg body weight led to remarkable new endosteal bone formation [65]. Localized application of TGF-β1 antibody resulted in delayed and impaired endochondral bone formation during the healing of bone fractures [59]. According to the researches above, interestingly, the conflict results in vitro are not shown in in vivo studies, and there may be additional regulation mechanisms in in vivo conditions, which may require further investigation.

A possible reason for the conflicting results is that the concentration of TGF-β1 contributes to its effect on MSCs. Asparuhova et al. reported that a specific concentration of TGF-β1 promoted osteogenic gene expression of primary extraction socket tissue cells but a higher concentration of TGF-β1 inhibited osteogenic gene expression [66]. Xu et al. reported a dual role of TGF-β1 in osteogenic differentiation of mouse BMSCs in vitro. Adding 1 ng/mL TGF-β1 promoted osteogenic differentiation, as increased *ALP* and *Osterix* expression indicated. However, 10–50 ng/mL TGF-β1 inhibited osteogenic differentiation of BMSCs in a dose-dependent manner. In addition, 100 ng TGF-β1 in 250 µL Hydrogel promoted the healing of calvarial bone defects in nude mice, but 2 µg TGF-β1 inhibited the healing process [55]. Therefore, there is likely an optimal concentration of TGF-β1 that promotes osteogenesis, and higher concentration of TGF-β1 inhibits osteogenic differentiation of MSCs instead. However, in the in vitro studies mentioned above, it is difficult to compare the concentrations of TGF-β1, because methods to induct TGF-β1 in MSCs are distinct. Besides, it is difficult to measure local TGF-β1 concentrations in vivo, especially for systematic administration [16, 54, 65]. Furthermore, the tendency to promote bone formation by TGF-β1 in vivo may result from a lower local concentration of TGF-β1 in comparison to in vitro studies. Therefore, the correlation between concentration of TGF-β1 and its regulation of cell fate determination of MSCs may provide an explanation for disputes in published studies, and the optimal concentration of TGF-β1 to promote osteogenic differentiation of MSCs under different in vivo condition may require further investigation.

### TGF-β2 and TGF-β3 regulate MSC functions

There are fewer studies of how TGF-β2 and TGF-β3 regulate the osteogenic differentiation of MSCs than TGF-β1; however, the findings are still contradictory.

TGF-β2 has been shown to inhibit osteogenic differentiation in in vitro studies. Tomoya et al. showed that TGF-β2 inhibited osteoblast differentiation of primary neonatal calvarial cells from mice [14]. In other studies, the overexpression or addition of TGF-β2 inhibited osteogenic differentiation of human BMSCs, and applying TGF-β2 antibody negated the effect [54, 56]. However, whether TGF-β2 induces or inhibits bone formation in vivo seems controversial, but more studies displayed increased bone formation of TGF-β2 in vivo. TGF-β2 in conjunction with collagenous matrix and porous hydroxyapatites induced increased heterotopic bone formation in rectus abdominis of baboons, but limited regeneration of calvarial defects in baboons were induced instead [60]. Dean et al. reported that addition of TGF-β2 in PFF (poly (propylene fumarate)) improved new bone amount and biomechanical strength in cranial defect of rabbits. Besides, a lower molecular weight PFF combined with TGF-β2 induced increased new bone formation synergistically, which indicated proper scaffold may improve bone formation effect of TGF-β2 in vivo [61]. According to results mentioned above, TGF-β2 displays different effects on cell fate determination of MSCs in contrast to TGF-β1, which indicates that distinct intracellular pathway may be activated by TGF-β1 and TGF-β2 in MSCs, although their structures are similar. However, TGF-β2 tends to promote bone formation in vivo in contrast to the results of in vitro studies. This indicates possible additional factors in vivo that modulate effects of TGF-β2 on osteogenic differentiation, which may require further investigation in future.

TGF-β3 inhibits osteogenic differentiation of human MSCs, and higher concentrations exhibit stronger inhibitory effects. Such conclusions have been supported by ALP and von Kossa staining of human MSCs incubated with TGF-β3-encapsulated microspheres at concentrations of 0, 0.035, 0.135, and 1.35 ng/mL [57]. However, overexpressing TGF-β3 in BMSCs of rats through lentivirus induces osteogenic differentiation, proven by ALP activity and expression of *Osteopontin* (*Opn*), *Osteocalcin* (*Ocn*), and *Osteoprotegerin* (*Opg*) [58]. These results indicate that a relatively high dosage of recombined human TGF-β3 induces rapid bone formation in baboons. Distinct effects of TGF-β3 on bone formation in other species have also been reported [67]. Therefore, differences among species may cause differences in the regulation of osteogenic differentiation by TGF-β3, which is more significantly revealed in comparison to TGF-β1 and TGF-β2. Additionally, this also indicates possibly diverse intracellular response to TGF-β3 of MSCs.

### Mechanisms of TGF-β signaling underlying the regulation of osteogenic differentiation of MSCs

Osteogenic differentiation is an essential cell fate determination for MSCs. Therefore, the mechanisms of TGF-β signaling underlying the regulation of gene expression related to osteogenesis are discussed here, which is displayed in Fig. 8. The mechanism of how TGF-β1 regulates osteogenic differentiation of MSCs has been studied as follows. RUNX2 is an essential transcription factor for the proliferation of osteoprogenitor cells, osteogenic differentiation, and activation of genes responsible for osteogenic differentiation at multiple stages [68, 69]. Lee et al. found that *Runx2* was essential in the response to TGF-β1 and BMP-2 in inducing osteogenesis differentiation in the C1C12 mouse pluripotent mesenchymal precursor cell line. TGF-β1 or BMP signaling induces RUNX2 to bind to the DNA sequence and induces the expression of the type I collagen gene and *fibronectin*. In addition, RUNX2 itself is insufficient for the expression of osteoblast-specific genes such as *Ocn* and *Alp*. SMAD5 and RUNX2 cooperate to induce *Alp* expression and activity. *Runx2* is not a direct target of TGF-β signaling [70]. *Junb* is directly induced by SMAD and is located upstream of RUNX2, and p38 MAPK also induces RUNX2 activation by TGF-β1 [71]. Interaction between RUNX2 and JUNB induces collagenase-3 expression in human breast cancer cells, and SMAD3 induced by TGF-β1 is required to stabilize the complex [72]. However, it is unknown whether a similar mechanism exists in the induction of osteogenic genes of TGF-β1. BMP2 is another TGF-β superfamily factor that promotes osteogenesis. A lower dosage of TGF-β1 activates SMAD3 to bind to the *Bmp2* promoter to upregulate the expression of *Bmp2*, thus promoting osteogenic differentiation of MSCs. A higher dosage of TGF-β1 changes the binding site of SMAD3 in the *Bmp2* promoter and inhibits its expression. Higher TGF-β1 also increases tomoregulin-1, thus repressing the expression of *Bmp2* and osteogenic differentiation. Therefore, the different dosages of TGF-β1 are responsible for its dual role in osteogenic differentiation of MSCs, and this affects BMP2, another important factor in osteogenesis [55]. However, TGF-β1 activates the MAPK pathway and induces phosphorylation of RUNX2, thus suppressing *Ocn* promotor activity [53]. TGF-β1 also promotes matrix maturation but inhibits its mineralization during osteogenic differentiation, as seen by increased ALP and collagen staining but downregulated *Ocn* promotor. TGF-β1 transcriptionally induces SMAD ubiquitination regulatory factor 1 (SMURF1) to promote the degradation of CCAAT/enhancer binding protein beta (C/EBPβ). C/EBPβ can bind to the promoter of Dickkopf1 (DKK1), which is necessary for mineralization of the matrix by osteoblasts. TGF-β1 dramatically inhibits this process [73, 74]. According to researches above, both canonical and non-canonical TGF-β signaling are involved in regulation of osteogenic differentiation of MSCs by TGF-β1, and crosstalk within TGF-β superfamily members may provide a new way to investigate their roles in determination of MSCs fate. However, the mechanisms underlying dual role of TGF-β1 in expression of *Runx2* and *Ocn*, as shown in in vitro studies, seems to require further investigation [53, 54]. Therefore, there may be still unrevealed mechanisms of how TGF-β1 regulates cell fate determination of MSCs, although there have been abundant researches in this topic.

**Fig. 7:**
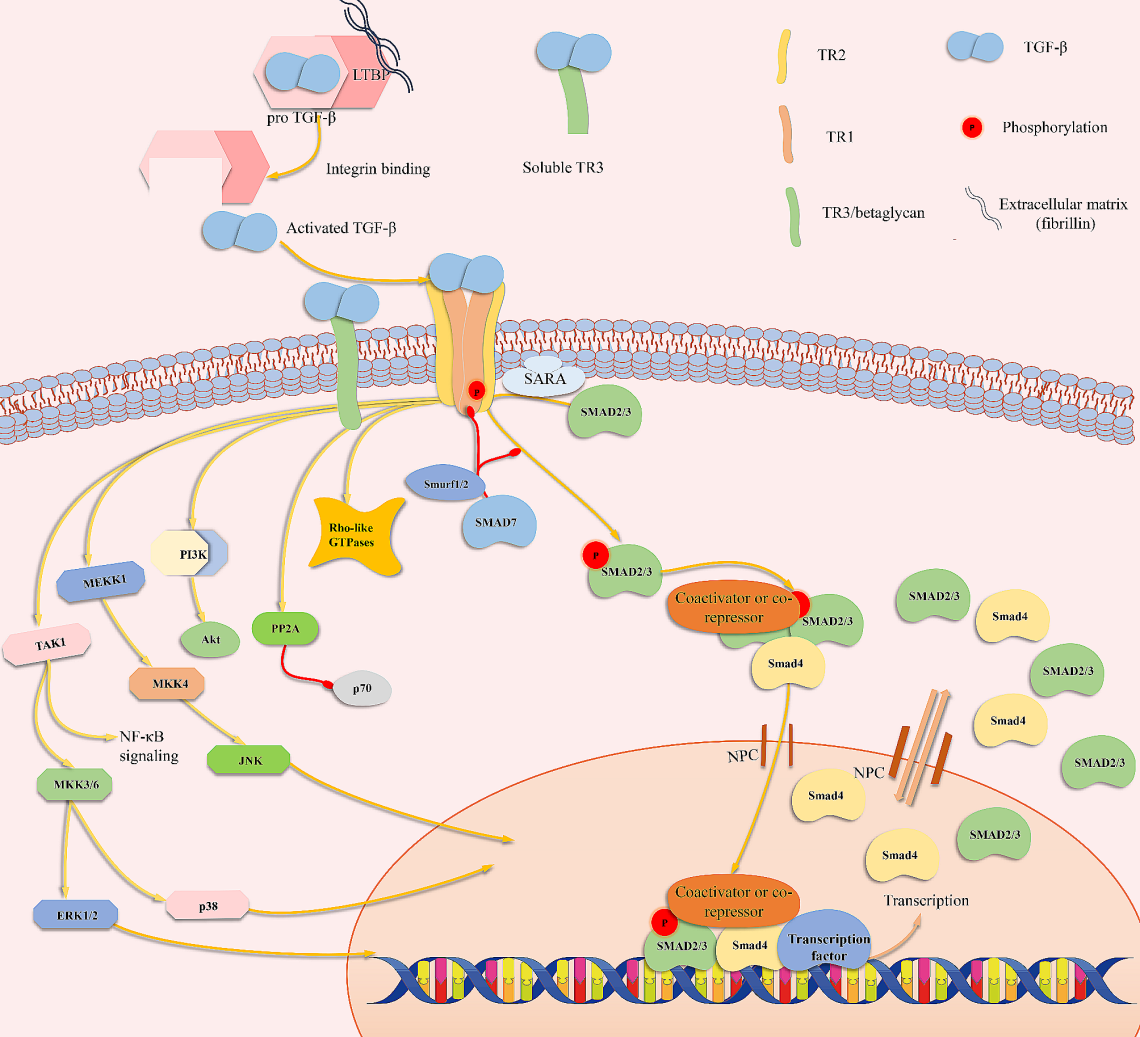
Mechanisms of TGF-β signaling. TGF-βs are secreted growth factors that function by binding to receptors on the cytomembrane. Latent TGF-β must be activated before binding to the receptors. SMADs and other intracellular proteins such as MKKKs, PI3K, and Rho-like GTPases can be activated by TGF-β signaling mainly through phosphorylation by TR1 (red circles), thus activating different signaling pathways. As a result, transcription factors are activated or repressed, the expression of specific genes is regulated, and the functions of cells are changed.

**Fig. 8:**
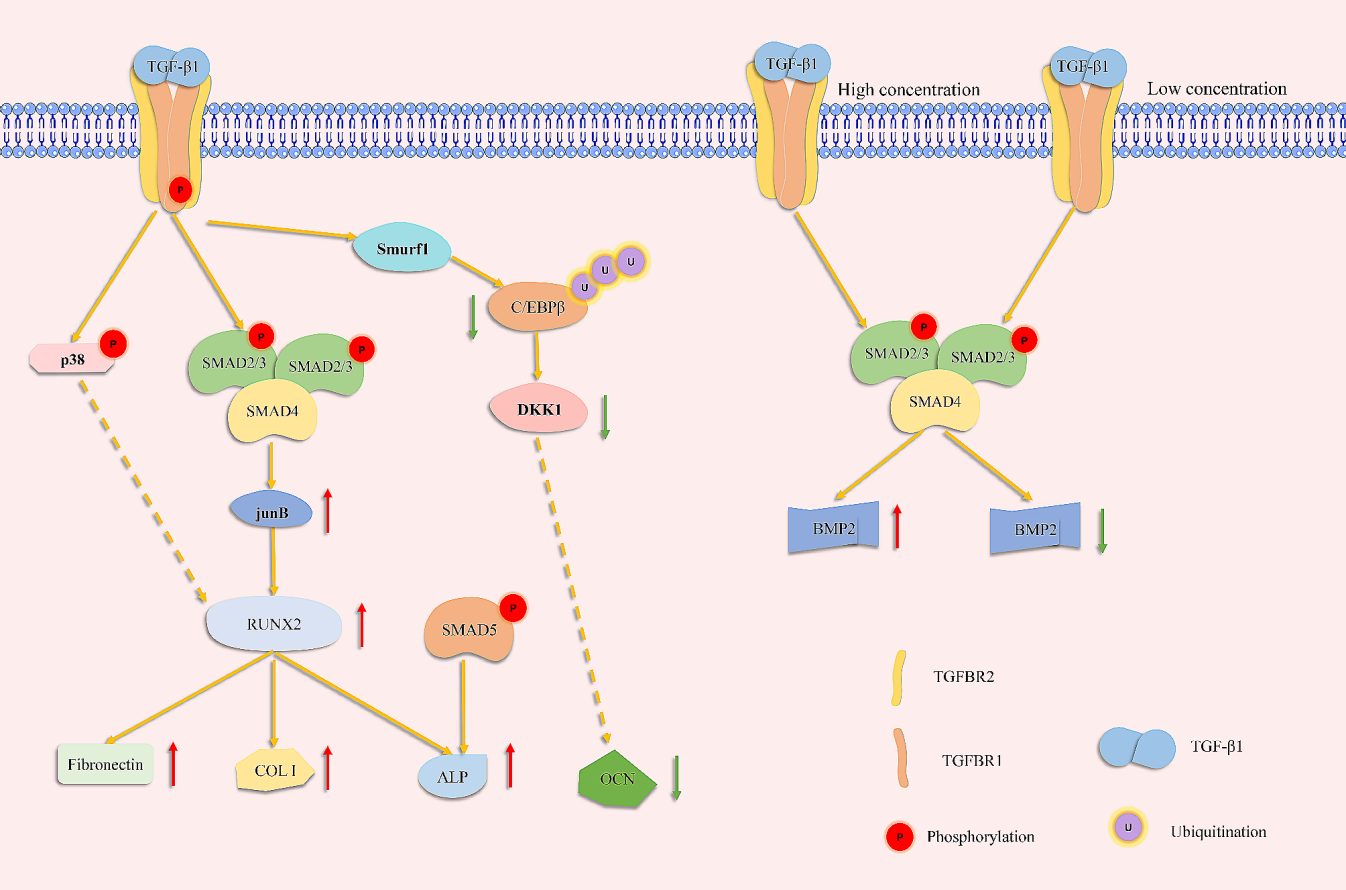
Mechanisms underlying how TGF-β1 regulates osteogenic differentiation. TGF-β1 induces expression of Runx2 through SMADs and p38, and RUNX2 is sufficient to induce *fibronectin* and type I collagen. SMAD5 induced by BMP signaling is needed to induce *Alp* expression. However, expression of *Ocn*, essential for matrix mineralization, is suppressed by TGF-β1. Distinct dosages of TGF-β1 influence the osteogenesis of MSCs by regulating BMP2 expression.

In comparison to TGF-β1, the mechanisms for TGF-β2 and TGF-β3 are less explored. Sun et al. reported that treatment with TGF-β2 activates extracellular signal-regulated kinases (ERKs) signaling in C3H10T1/2 mesenchymal pluripotent cells and inhibits early osteogenic differentiation by inducing the E3 ubiquitin-protein ligase SMURF1, leading to the degradation of RUNX2 [75]. Interestingly, SMURF1 activated by TGF-β1 are shown to inhibit mineralization during osteogenic differentiation of MSCs through inducing degradation of C/EBPβ [73, 74]. The underlying mechanism of diverse effects of SMURF1 induced by TGF-β1 and TGF-β2 is an interesting topic, which may require further research.

As long as we known, the distinct mechanisms of regulation of osteogenic differentiation of MSCs by three TGF-β isoforms are still not clearly clarified in present, which are promising directions for future researchers to explore. TGF-β superfamily members modulate multiple biological process, and the mechanisms of contextual determinants affecting their action in embryo development, immunity and tumor progress have been comprehensively reviewed [1]. These findings may inspire potential directions for investigations in the fate determination of MSCs. Firstly, the access of TGF-β ligands for the receptors can be regulated by several factors. Extra cellular matrix is a platform for activation and modulation of TGF-β ligands [2]. Membrane dynamics is also proved to modulates the distribution of other superfamily members [1]. Co-receptors, such as Betaglycan, may modulate the binding ability of three ligands distinctly [3]. The differences in factors mentioned above may provide possible explanation to controversial currently, especially for inconsistent findings in vivo and in vitro. Besides, distinct intracellular responses to TGF-β isoforms in MSCs are not fully clarified, especially for TGF-β2 and TGF-β3. The gene sites for SMAD complex to binding is distributed genome wide. Therefore, to achieve specific cellular response to TGF-β induced SMAD activation, there needs to be other transcription factors activated by other cytokines or intrinsic for cells [1]. The transcription factors that play synergistic roles with SMAD2/3 in RUNX2 expression induced by TGF-β1 has been investigated [4, 5], but the researches into TGF-β2 and TGF-β3 still lack as long as we know. There is also non-canonical signaling of TGF-β, in which transcription factors other than SMADs are activated. Whether TGF-β isoforms activate the multiple intracellular pathway distinctly in MSCs and the underlying mechanisms are possibly an interesting topic for future researches.

The three TGF-β isoforms have distinct roles in osteogenic differentiation in vitro and in vivo, as summarized in Tables 1 and 2. The different treatment concentrations and species may partially explain the contradictions among the published studies on this topic. The mechanisms underlying how TGF-β signaling regulates genes related to osteogenesis have been investigated; however, how TGF-β1 regulates osteogenic differentiation through noncanonical signaling and how TGF-β2 and TGF-β3 regulate osteogenic differentiation of MSCs are not well known. Therefore, differences between species and control conditions may paly essential role in response of MSCs to TGF-β signaling. Additionally, there are still gaps in known mechanisms of cell fate determination of MSCs regulated by TGF-β signaling.

**Table 1:** TGF-β isoforms regulate osteogenic differentiation of MSCs in vitro

Cell type	TGF-β isoform	Method	Influence on osteogenic differentiation	Test method	Mechanism	Reference
Human BMSCs	TGF-β1	Lentiviral overexpression	Promotion	RT-PCR and WB of OCN and RUNX2	-	[54]
	TGF-β2	Lentiviral overexpression	Inhibition	RT-PCR and WB of OCN and RUNX2		
Rat differentiating osteoblast	TGF-β1	Treatment with human TGF-β1	Inhibition; decreased *Ocn* expression but unchanged *Runx2* expression	RT-PCR of OCN and RUNX2	Increasing phosphorylation of RUNX2 through MAPK pathway	[53]
MPCs from 3-month and 9-month old mice	TGF-β1	Treatment with TGF-β1	Inhibition	ALP staining	Increasing degradation of TRAF3 and promoting GSK-3β-mediated degradation of β-catenin	[16]
Primary ESsT-Cs	TGF-β1	Treatment with 0, 1, 5 ng/mL of TGF-β1	1 ng/mL TGF-β1: promotion; 5 ng/ml TGF-β1: inhibition	RT-PCR of COL1A1, SPP 1, RUNX2, ALPL. DLX5, IBSP, BGLAP2, PHEX; ARS	-	[66]
Mouse BMSCs	TGF-β1	Treatment with 1–50 ng/mL TGF-β1	1ng/mL TGF-β1: promotion; 10-50ng/mL TGF-β1: inhibition	RT-PCR of ALP, OSX, RUNX2, OCN, COL1; ALP staining	Low concentration of TGF-β1 activates SMAD3 and promoted their binding with *Bmp2* promoter; high concentration of TGF-β1 increase tomoregulin-1 and represses *Bmp2*	[55]
Mouse primary calvarial cells	TGF-β2	Treatment with 1ng/mL TGF-β2	Inhibition	ARS	-	[14]
Young and old CD271^+^SSEA-4^+^ human BMSCs	TGF-β2	Treatment with 100 ng/mL TGF-β2 or 10 µM/mL 11D1, anti-TGF-β monoclonal antibody	TGF-β2: inhibition; 11D1: promotion	Calcein staining	-	[56]
Human BMSCs	TGF-β3	Incubated with TGF-β3 encapsulated PGLA microspheres at concentration of 0, 0.035, 0.135, 1.35 ng/mL	Inhibition	ALP staining and von Kossa staining	-	[57]
Rat BMSCs	TGF-β3	Lentiviral overexpression	Promotion	ALP activity; RT-PRC of OPG and OPN	-	[58]

A brief list of in vitro studies investigating three TGF-β isoforms regulates osteogenic differentiation of MSCs

BMSCs bone merrow stromal cells, ESsT-Cs extraction socket soft tissue cells, OCN osteocalcin, RUNX2 runt-related transcription factor 2, ALP alkaline phosphatase, OSX Osterix, OPN osteopotin, ARS alizarine red staining

**Table 2:** TGF-β isoforms regulate osteogenic differentiation of MSCs in vivo

Species	Preparation	Dose	Method of administration	Duration	Test method	Influence on bone formation	Reference
Rats and rabbits	Human recombinant TGF-β1	1000 µg/kg body weight	Intravenous injection	5 days	HE staining	Endosteal new bone formation	[65]
Mice	TGF-β1 neutralizing anti-body	-	Subcutaneous injection at the fracture site	10, 14 and 21 days	µCT, ABH staining	Impaired endochondral bone formation	[59]
Mice	BMSCs mixed with 45 mg of β-TCP	100ng, 200ng, 1 µg, 2 µg TGF-β1 in 250µL of Hydrogel	Subcutaneous implantation	8 weeks	HE staining; OCN immunochemistry; RT-PCR of ALP and OCN	100 ng and 200 ng of TGF-β1: promoting bone formation; 2 µg of TGF-β1: inhibiting bone formation	[55]
Baboon (*Papio ursinus*)	Human recombinant TGF-β2	1,5,25 µg in the rectus abdominis; 10–100 µg in the calvarial defect	Implantation with collagenous matrix and sintered hydroxyapatite in the calvarial defect and the rectus abdominis	30 and 90 day	Goldner’s trichrome staining	Increased heterotopic bone formation in the rectus abdominis but limited calvarial bone regeneration	[60]
New Zealand White rabbits	TGF-β2	0.8 µg TGF-β2 for each PFF implantation	Implantation with PFF with β-TCP scaffold in cranial defect	6 weeks and 12 weeks	Toluidine blue staining and biochemical push-in tset	Improved bone amount and quality	[61]

A brief list of in vivo studies investigating three TGF-β isoforms regulates osteogenic differentiation of MSCs

β-TCP β-tricalcium phosphate, HE staining Hematoxylin and eosin staining, ABH staining Alcian Blue Hematoxylin staining, PFF poly(propylene fumarate)

## Conclusion

TGF-β signaling is essential to many biological processes, especially in the process of bone metabolism. MSCs are stem cells with osteogenic differentiation and multiple tissue resources, and MSCs has drawn much attention in regard to bone disease treatment. Distinct role of three TGF-β isoforms in osteogenic differentiation of MSCs are summarized in this review. According to the published studies, the conclusions below may be drawn. (1) There is an optimal concentration of TGF-β1 that promotes osteogenic differentiation of MSCs, and higher concentrations inhibit the process. Therefore, it is proposed that the controversial conclusions of published studies mainly result from the uncontrolled concentration of TGF-β1. (2) The roles of TGF-β2 and TGF-β3 in regulation of MSCs osteogenic differentiation are less explored in contrast to TGF-β1, but according to published studies, the three isoforms of TGF-β play distinct role in osteogenic differentiation of MSCs. This provide a possible explanation for conflict conclusions in researches that do not investigate roles of the three isoforms in MSCs separately. Further and systematic investigations are still required. (3) Systematic exploration on the optical concentration of TGF-β1 to promote osteogenic differentiation is still required for further application in vivo.

Disruption of TGF-β signaling leads to human genetic diseases or acquired diseases related to defects in bone tissue and dysregulations in the bone repair process. For prospect, TGF-β targeted therapies may be promising for bone diseases. There is phase I clinical trial demonstrating that fresolimumab, a TGF-β neutralizing antibody, effectively increases bone mass and is well tolerated in osteogenesis imperfecta patients [12]. Therapeutic approaches to TGF-β have developed for years and there are products put into clinical use or clinical trials [76]. Therefore, TGF-β targeted therapy may be promising solutions for bone diseases and the cost of new drug development and safety confirmation can be saved. Considering the functions of the TGF-β isoforms separately will aid our understanding of diseases related to TGF-β signaling and provide possible targets for potential clinical applications. Further investigation into TGF-β signaling will reveal its additional functions and more detailed mechanisms and will aid our knowledge of its functions in bone homeostasis.

### Electronic supplementary material

Below is the link to the electronic supplementary material.

Supplementary Material 1

## Data Availability

Not applicable.

## Abbreviations

ALP: Alkaline phosphatase
ARS: Alizarine red staining
AMH/MIS: Anti-Müllerian hormone/Müllerian inhibiting substances
BMP: Bone morphogenic protein
BMSCs: Bone marrow stromal cells
CBP: CREB binding protein
C/EBPβ: CCAAT/enhancer binding protein beta
DKK1: Dickkopf1
ER: Endoplasmic reticulum
ERK: Extracellular signal-regulated kinases
GDF: Growth differentiation factor
GS domain: Glycine- and serine-rich domain
I-SMADs: Inhibitory SMADs
JNK: C-Jun amino-terminal kinases
LAP: Latent associated peptide
LTBPs: Latent TGF-β binding proteins
MAPKs: Mitogen-activated protein kinases
MKK: MAPK kinase
MKKK: MAPKK kinase
MKK3: MAPK kinase 3
MKK6: MAPK kinase 3
mTOR: Mammalian target of rapamycin
MPCs: Mesenchymal progenitor cells
MSCs: Mesenchymal stem cells
Msx2: *Msh* homeobox 2
OCN: Osteocalcin
OPG: Osteoprotegerin
OPN: Osteopontin
OSX: Osterix
PP2A: Protein phosphatase 2 A
PI3K: Phosphatidylinositol-3-kinase
R-SMADs: Receptor-activated SMADs
RUNX2: Runt-related transcription factor 2
SMAD: *Sma* and *Mad* homolog
SMURF1: SMAD ubiquitination regulatory factor 1
TAK1: TGF-β-activated kinase 1
TGF-β: Transforming growth factor β
TR1: TGF-β receptor 1
TR2: TGF-β receptor 2
TR3: TGF-β receptor 3
XIAP: X-linked inhibitor of apoptosis protein
