# Supplementary material for: TGF-β signaling regulates differentiation of MSCs in bone metabolism: disputes among viewpoints
Source: Stem Cell Res Ther. 2024 May 31;15:156. doi: 10.1186/s13287-024-03761-w (PMC11140988; doi:10.1186/s13287-024-03761-w)
Supplement: Supplementary file 1 — Supplementary Material 1 [file 13287_2024_3761_MOESM1_ESM.docx]

**Supplementary appendix**

**Appendix 1：**The searching words for role of TGF-β1,2,3 in MSCs osteogenic differentiation *in vitro* and *in vivo* separately are as follows:

(((((((((((("Transforming Growth Factor beta1"[Mesh]) OR (Transforming Growth Factor beta I)) OR (TGF-beta1)) OR (Transforming Growth Factor-beta1)) OR (TGF-beta-1)) OR (TGF beta 1)) OR (Transforming Growth Factor beta 1 Latency Associated Peptide)) OR (TGF-beta1 Latency-Associated Protein)) OR (Latency-Associated Protein, TGF-beta1)) OR (TGF beta1 Latency Associated Protein)) OR (TGF-beta1LAP)) OR (TGF beta1LAP))AND(((((((((((("Osteogenesis"[Mesh]) OR (Bone Formation)) OR (Ossification)) OR (Ossifications)) OR (Endochondral Ossification)) OR (Endochondral Ossifications)) OR (Ossification, Endochondral)) OR (Ossifications, Endochondral)) OR (Physiologic Ossification)) OR (Ossification, Physiological)) OR (Physiological Ossification)) OR (Ossification, Physiologic))

((((((((((((((((("Transforming Growth Factor beta2"[Mesh]) OR (Glioblastoma-Derived T-Cell Suppressor Factor)) OR (Glioblastoma Derived T Cell Suppressor Factor)) OR (Cartilage-Inducing Factor-B)) OR (Cartilage Inducing Factor B)) OR (Polyergin)) OR (TGF-beta2)) OR (TGF-beta-2)) OR (BSC-1 Cell Growth Inhibitor)) OR (BSC 1 Cell Growth Inhibitor)) OR (Suppressor Factor, T-Cell, Glioblastoma-Derived)) OR (Transforming Growth Factor beta 2 Latency Associated Peptide)) OR (TGF-beta2LAP)) OR (TGF beta2LAP)) OR (TGF-beta2 Latency-Associated Protein)) OR (Latency-Associated Protein, TGF-beta2)) OR (TGF beta2 Latency Associated Protein))AND(((((((((((("Osteogenesis"[Mesh]) OR (Bone Formation)) OR (Ossification)) OR (Ossifications)) OR (Endochondral Ossification)) OR (Endochondral Ossifications)) OR (Ossification, Endochondral)) OR (Ossifications, Endochondral)) OR (Physiologic Ossification)) OR (Ossification, Physiological)) OR (Physiological Ossification)) OR (Ossification, Physiologic))

((((((((((("Transforming Growth Factor beta3"[Mesh]) OR (TGF-beta-3)) OR (TGF beta 3)) OR (TGFB3)) OR (TGF-beta3)) OR (TGF beta3)) OR (Transforming Growth Factor beta 3 Latency Associated Peptide)) OR (TGF-beta3 Latency-Associated Protein)) OR (Latency-Associated Protein, TGF-beta3)) OR (TGF beta3 Latency Associated Protein)) OR (TGF-beta3LAP))AND(((((((((((("Osteogenesis"[Mesh]) OR (Bone Formation)) OR (Ossification)) OR (Ossifications)) OR (Endochondral Ossification)) OR (Endochondral Ossifications)) OR (Ossification, Endochondral)) OR (Ossifications, Endochondral)) OR (Physiologic Ossification)) OR (Ossification, Physiological)) OR (Physiological Ossification)) OR (Ossification, Physiologic))
